# Neuroprotective and antioxidative effects of pioglitazone in brain tissue adjacent to the ischemic core are mediated by PI3K/Akt and Nrf2/ARE pathways

**DOI:** 10.1007/s00109-021-02065-3

**Published:** 2021-04-16

**Authors:** Yi Zhao, Ulf Lützen, Peter Gohlke, Ping Jiang, Thomas Herdegen, Juraj Culman

**Affiliations:** 1 Department of Nuclear Medicine, Molecular Imaging, Diagnostics and Therapy, University Hospital of Schleswig-Holstein, Campus Kiel, 24105 Kiel, Germany; 2 Institute of Experimental and Clinical Pharmacology, University Hospital of Schleswig-Holstein, Campus Kiel, 24105 Kiel, Germany; 3 University Clinic for Medical Radiation Physics, Medical Campus Pius-Hospital, Carl von Ossietzky University Oldenburg, 26121 Oldenburg, Germany; 4 Institute of Experimental and Clinical Pharmacology, Faculty of Medicine, University Hospital of Schleswig-Holstein, Campus Kiel, Arnold Heller Strasse 3, 24105 Kiel, Germany

**Keywords:** Neuroprotection, Nrf2, PPARγ, Pioglitazone, PI3K/Akt, Cerebral ischemia

## Abstract

**Abstract:**

The present study elucidates the neuroprotective mechanisms of the PPARγ (peroxisome proliferator-activated receptor γ) agonist pioglitazone in survival of ischemic neurons following middle cerebral artery occlusion with reperfusion (MCAO). Intracerebroventricular infusion of pioglitazone over 5 days before and 24 or 48 h after MCAO alleviated neurological impairments, inhibited apoptosis 24 h, and activated the PI3K/Akt pathway along with increased phosphorylation of Akt (ser473) and GSK-3β (ser9) in the peri-infarct cortical areas 48 h after MCAO. In primary cortical neurons, pioglitazone suppressed the glutamate-induced release of lactate dehydrogenase by a PPARγ-dependent mechanism. This protective effect was reversed after co-treatment with PI3K and Akt inhibitors, LY294002 and SH-6, respectively. Pioglitazone enhanced the expression of the antioxidative transcription factor Nrf2 and its target gene protein, heme oxidase-1, in the peri-infarct area. Pioglitazone also increased activation of the antioxidant response element (ARE) in neuronal PC12 cells transfected with the pNQO1-rARE plasmid. We demonstrate in primary cortical neurons from Nrf2 knockout mice that the lack of Nrf2 completely abolished the neuroprotective effects of pioglitazone against oxidative and excitotoxic damage. Our results strongly suggest that the neuroprotective effects of PPARγ in peri-infarct brain tissues comprise the concomitant activation of the PI3K/Akt and Nrf2/ARE pathways.

**Key messages:**

- Pioglitazone inhibits apoptosis in ischemic brain tissue. 
- Pioglitazone acting on PPARγ activates PI3K/Akt pathway in ischemic brain tissue.
- Pioglitazone activates via Nrf2 the antioxidant defense pathway in injured neurons.
- Pioglitazone activates the antioxidant response element in neuronal PC12 cells.
- Pioglitazone fails to protect primary neurons lacking Nrf2 against oxidative damage.
- Activation of PPARγ supports the survival of viable neurons in peri-infarct regions.

**Supplementary Information:**

The online version contains supplementary material available at 10.1007/s00109-021-02065-3.

## Introduction

Tissue damage following cerebral ischemia depends on the reduction of the cerebral blood flow and on the duration of the ischemic insult [1, 2]. Application of the recombinant tissue plasminogen activator or mechanical thrombectomy is the only therapeutic interventions in stroke patients. An effective and well-tolerated neuroprotective therapy is still not available [3]. The data of a meta-analysis of experimental studies in rodents subjected to cerebral ischemia suggests that peroxisome proliferator-activated receptor γ (PPARγ) agonists improve the recovery from ischemic stroke [4]. Moreover, the preclinical trial has demonstrated that systemic treatment with pioglitazone of rats in the post-ischemic phase reduces infarct volume, alleviates neurological impairments, inhibits neuronal degeneration, and improves the outcome after stroke [5].

The PPARγ is constitutively expressed in the brain and cerebral ischemia robustly upregulates the PPARγ gene expression in neurons. The increased PPARγ expression in the peri-focal cortical areas is further enhanced by pioglitazone [6, 7]. PPARγ deficiency in neurons increased their susceptibility to ischemic brain damage [8]. These findings point to the unique function of *cerebral* PPARγ in ischemic brain tissue. A few studies focused on the effects of brain PPARγ activation on inflammatory reactions in ischemic brain tissue [7, 9, 10]. However, the exact role of cerebral PPARγ in the neuroprotection promoted by pioglitazone has not yet been extensively studied. Therefore, we investigated the effect of pioglitazone acting on cerebral PPARγ on activation of the canonical survival antioxidative phosphoinositide 3-kinase (PI3K)/Akt (protein kinase B)- and nuclear factor erythroid 2-related factor (Nrf2)/ARE pathways.

The death cascades activated by ischemic injury are counteracted by survival signals, such as activation of the PI3K/Akt pathway which inhibits apoptosis and promotes neuroprotection. Glycogen synthase kinase-3β (GSK-3β) is an essential enzyme downstream to Akt. Its phosphorylation at ser9 by Akt shifts the enzyme to its inactive conformation and prevents thus its pro-apoptotic function [11].

The Nrf2 is the major mediator of cellular defense reactions against oxidative stress. Exposure to high concentrations of reactive oxygen species (ROS), phosphorylation of serine/threonine residues of Nrf2, or inactivation of GSK-3β disrupts the Kelch-like ECH-associated protein 1 (Keap1)/Nrf2 complex in the cytoplasm. Nrf2 translocates into the nucleus and triggers the transcription of numerous genes encoding for antioxidant enzymes [12].

In the present study, we investigated the effects of intracerebroventricularly (ICV) infused pioglitazone on the expression of apoptosis markers and activation of individual components of the PI3K/Akt pathway and Nrf2/heme oxidase-1 (HO-1) in the peri-infarct frontoparietal cortex following the occlusion of the middle cerebral artery (MCAO). The role of PI3K, Akt, and Nrf2 in the pioglitazone-mediated neuroprotection against excitotoxicity and oxidative damage was studied in primary cortical neurons obtained from rats and Nrf2 knockout mice.

## Materials and methods

### Animals

Male Wistar rats 9–10 weeks old were obtained from Charles River (Sulzfeld, Germany). The rats were housed in the animal facility which meets the requirements of the European Directive 2010/63/EU on the Protection of Animals Used for Scientific Purposes. Zero- to 1-day neonatal Wistar rats or 0 to 1 neonatal C57BL/6 mice and Nrf2 knockout mice (B6.129X1-*Nfe2l2*^*tm1Ywk*^/J) were obtained from the same animal facility. All efforts have been made to minimize the suffering of the animals.

### Surgical procedures (MCAO)

Osmotic minipumps (ALZET Model No. 2002, Charles River, Sulzfeld, Germany) filled with vehicle (50% dimethyl sulfoxide in physiological saline) or with pioglitazone (6 mmol/l, Takeda Pharma, Japan) were implanted 5 days before MCAO for 90 min with subsequent reperfusion [13]. In sham-operated rats, the occluding filament was not inserted. Regional cerebral blood flow (rCBF) was continuously monitored in each rat at one point (1 mm posterior to the bregma, 6 mm from the midline) on the surface of the hemisphere by laser-Doppler flowmetry (Periflux system 5000; PERIMED AB, Järfälla, Sweden). Body temperature was maintained at 37 °C with a heating pad. Chloral hydrate (400 mg/kg body weight) injected intraperitoneally was used as anesthetic for all surgical procedures. Rats in which no increase in rCBF was detected after pulling out the monofilament or rats suffering from intracerebral hemorrhage were excluded from the experimental protocols.

### Neurological assessment

Neurological evaluations [14] were carried out by a blinded observer 24 and 48 h after MCAO as reported in detail previously [5] (sham-operated *n*=10, vehicle-treated *n*=12–14, and pioglitazone-treated *n*=12–14 at each time point). The minimum of the total neurological score is 3 and the maximum is 18 (see the Supplement).

### Primary neuronal cell culture

Cortical neuronal cell cultures were prepared from the cerebral cortices from 0- to 1-day neonatal Wistar rats and from 0- to 1-day neonatal C57BL/6 mice (controls) or Nrf2 knockout mice as described previously [9, 15] (see the Supplement).

### Experimental protocols

- Experiment 1: Western blot analysis of apoptosis markers and individual components of the PI3K/Akt pathway and Nrf2/HO-1

On day 6 after the implantation of osmotic minipumps, vehicle- and pioglitazone-treated rats were exposed to MCAO. The brains were removed from the skull 24 h (vehicle, *n* = 8; pioglitazone, *n* = 8, sham-operated, *n* = 8) or 48 h (vehicle, *n* =8; pioglitazone, *n* = 8; sham-operated, *n* = 8) after MCAO, placed on the dorsal surface into a plastic brain matrix with a coronal slice orientation (World Precision Instruments, Sarasota, FL, USA), and cut into serial 1-mm-thick slices. Under a microscope, 1-mm-wide (determined from the infarct margin) pieces of frontoparietal cortical tissue were dissected from 3 consecutive coronal brain sections as described in detail previously [7]. The isolated proteins were used for Western blotting (see the Supplement).

- Experiment 2: Immunofluorescence staining for phospho-Akt (p-Akt) (ser473), phospho-GSK-3β (p-GSK-3β) (ser9), PPARγ and Nrf2 in neurons localized at the border of the infarct region

Twenty-four or 48 h after MCAO, vehicle-treated (*n*=5) or pioglitazone-treated (*n*=5) rats were deeply anesthetized and intracardially perfused with ice-cold phosphate-buffered saline (pH 7.4) followed by 4% paraformaldehyde. Brain coronal sections (10 μm) were used for immunofluorescence staining [9] (see the Supplement).

- Experiment 3: Effects of pioglitazone acting on PPARγ on glutamate-induced cytotoxicity in rat primary cortical neurons. The role of the PI3K and Akt

Seven days after plating, primary cortical neurons were treated with vehicle or pioglitazone (10 μM) in the presence or absence of the PPARγ antagonist GW 9662 (1 μM) added to the medium 30 min prior to pioglitazone. Thirty minutes after vehicle or pioglitazone treatment, cells were exposed to glutamate (100 μM). The concentrations of glutamate, pioglitazone, and GW 9662 were determined in previous experiments [15]. The assessment of cellular toxicity, carried out 24 h after glutamate treatment, was based on the quantification of lactate dehydrogenase (LDH) activity released from the cytosol of damaged cells into the culture medium (Cytotoxicity Detection Kit, Roche Diagnostics) according to the manufacturer’s recommendations.

In the second set of experiments, primary cortical neurons were treated with vehicle or pioglitazone and exposed to glutamate (100 μM). One set of cells was pre-treated with LY294002 (10 μM), a non-selective, reversible inhibitor of PI3Kα/δ/β with IC_50_ of 0.5/0.57/0.97 μM, respectively. LY294002 is by far the most frequently used PI3K inhibitor in the medical biology research. Another set of cells was pre-treated with the selective Akt inhibitor SH-6 (5 μM). The inhibitors were added to the medium 30 min prior to pioglitazone. LDH release into the medium was determined 24 h after the treatment. The exposure of cells to pioglitazone, GW 9662, LY294002, and SH-6 alone did not induce any toxic effects (Fig. 4, and Table A in the Supplement). Each experiment was repeated 3 to 4 times with 3 samples in each group.

- Experiment 4: Induction of the antioxidant response element (ARE)-mediated reporter activity by pioglitazone

Transfection of PC12 rat pheochromocytoma cells and the dual luciferase assay were carried out according to the method reported by Wruck et al. [16] (see the Supplement). Twenty-four hours after the transfection, the cells were stimulated with pioglitazone (1, 5, and 10 μM) with or without the PPARγ antagonist GW 9662 (1 μM). The activities of both luciferases were determined 24 h after stimulation with the PPARγ ligands. The luciferase activities were normalized to the *Renilla reniformis* luciferase activity of the internal control.

- Experiment 5: Effects of pioglitazone on 6-hydroxydopamine (6-OHDA)- and glutamate-induced cytotoxicity in mouse primary cortical neurons from control and Nrf2 knockout mice

Seven days after plating, primary cortical neurons prepared from control, wild-type (C57BL/6) mice, and Nrf2 knockout mice were treated with vehicle or pioglitazone (10 μM) in the presence or absence of the PPARγ antagonist GW 9662 (1 μM). Thirty minutes after vehicle or pioglitazone treatment, neuronal cells were exposed to 6-OHDA (50 μM) or glutamate (100 μM) for 24 h. The neuronal cell death was assessed by measuring the LDH release to the medium. The immunofluorescence detection of β III-tubulin was carried out as described previously [15] (see the Supplement).

All experimental protocols were approved by the Governmental Committee for the Ethical Use of Experimental Animals in the German Federal State of Schleswig-Holstein and followed the Guidelines of the National Committee for the Protection of Animals Used for Scientific Purposes of the Federal Republic of Germany based on the Article 49 of EU Directive 2010/63/EU.

### Chemicals and reagents

Pioglitazone obtained from Takeda Pharmaceuticals (Japan) was dissolved in DMSO and ultra-pure water (1:1) (stock solution). GW 9662, a selective PPARγ antagonist (IC_50_ = 3.2 nM), was purchased from Cayman Chemicals Company and dissolved in DMSO. The vehicle solution consisted of DMSO and ultra-pure water. LY294002 (Cell Signaling Technology) and SH-6 (Enzo Life Sciences) were dissolved in DMSO (stock solution 10 mM) and diluted before the experiment with the culture medium. Glutamate (Sigma-Aldrich) was dissolved in the culture medium, and 6-OHDA (Sigma-Aldrich) in ascorbic acid (1 M) and diluted with the culture medium. All other chemicals and substances were purchased from Sigma-Aldrich or Merck.

### Statistical analyses

The values are expressed as means ± SD. The distribution of the data was analyzed by Kolmogorov-Smirnov’s test and Bartlett’s test was used to test the homogeneity of variances. The statistical evaluation of changes in rCBF was carried out by two-way analysis of variance (ANOVA) with repeated measures. Two-tailed *t*-test was used to compare the data on the quantitative analysis of immunofluorescence images (p-Akt and p-GSK-3β). One-way ANOVA, followed by a post hoc Bonferroni test for pairwise comparisons, or the Kruskal-Wallis test was used to compare the Western blot analyses of apoptotic and neuroprotection parameters, expression of Nrf2 and HO-1, and the data on the LDH release from primary cortical neurons and the dual luciferase assay. Statistical significance was accepted at *P* < 0.05.

## Results

### Regional cerebral blood flow, neurological outcome, and mortality

**Fig. 1:**
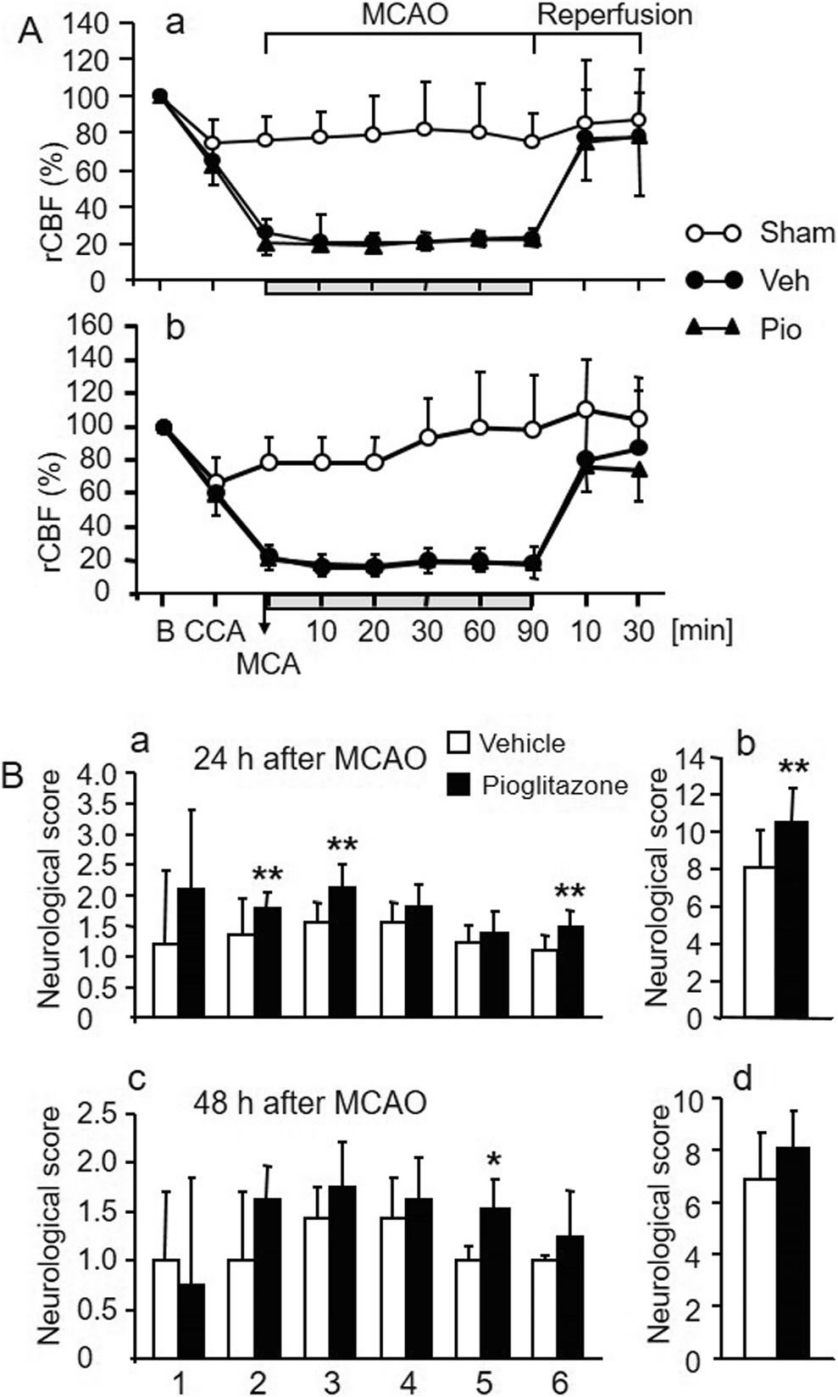
**A** Regional cerebral blood flow (rCBF) in the supply territory of the middle cerebral artery (MCA) in sham-operated animals (Sham), during occlusion of the MCA for 90 min (MCAO), and during reperfusion period in rats treated intracerebroventricularly (ICV) with vehicle (Sham and Veh) or pioglitazone (Pio). rCBF (mean ± SD) is expressed as a percentage of baseline values (B) (100%) recorded before occlusion of the common carotid artery (CCA). rCBF changes recorded in rats examined 24 h (a, upper panel) and 48 h (b, lower panel) after MCAO. Compared to the sham-operated rats, a significant drop in rCBF was recorded in rats exposed to MCAO (significance not shown). No differences between the vehicle-treated and pioglitazone-treated groups of rats were detected during MCAO and the reperfusion period. **B** Neurological scores of rats treated ICV with vehicle (empty columns) or pioglitazone (solid columns) and exposed to MCAO. a and b (upper panels): neurological scores 24 h; c and d (lower panels): 48 h after MCAO. The following neurological tests were evaluated: (1) spontaneous activity; (2) symmetry of the movement of four limbs; (3) forepaw outstretching; (4) climbing; (5) body proprioception; and (6) response to vibrissae touch. b and d: The summation of scores of motor deficits and sensory impairments. Rats with better neurological outcome received higher neurological scores. Neurological scores are expressed as the means ± SD. Statistical comparison to the vehicle-treated group: *P<0.05 and **P<0.01, calculated by Mann-Whitney test

The rCBF during MCAO and reperfusion period were identical in the vehicle-treated and the pioglitazone-treated groups of rats (Fig. 1A). During MCAO, rCBF dropped by 75–80% and recovered to 75–100 % of the baseline (B) in the reperfusion period. In sham-operated rats, rCBF decreased by 20–25 % during the first 30 min of the common carotid artery occlusion and completely regained the baseline values at later time points (Fig. 1A).

Moderate reductions of rCBF in sham-operated rats had no impact on neurological outcome. Twenty-four hours after MCAO, neurological scores of symmetry of the movements of 4 limbs, forepaw outstretching and vibrissae touch (Fig. 1B, a), and the summation of all neurological scores of rats treated with pioglitazone were significantly better than those of vehicle-treated rats (Fig. 1B, b). Forty-eight hours after MCAO, the pioglitazone-treated rats showed improved response to body proprioception (Fig. 1B, c). Previous findings have convincingly demonstrated that ICV infusion of pioglitazone reduces infarct volume and edema after ischemic stroke [7, 9, 10]. None of the sham-operated rats died during or after the surgery. Three rats treated with vehicle and 2 rats treated with pioglitazone died within 24 h after MCAO.

### Pioglitazone inhibits apoptotic cascade and promotes neuroprotection in the peri-infarct frontoparietal cortex

**Fig. 2:**
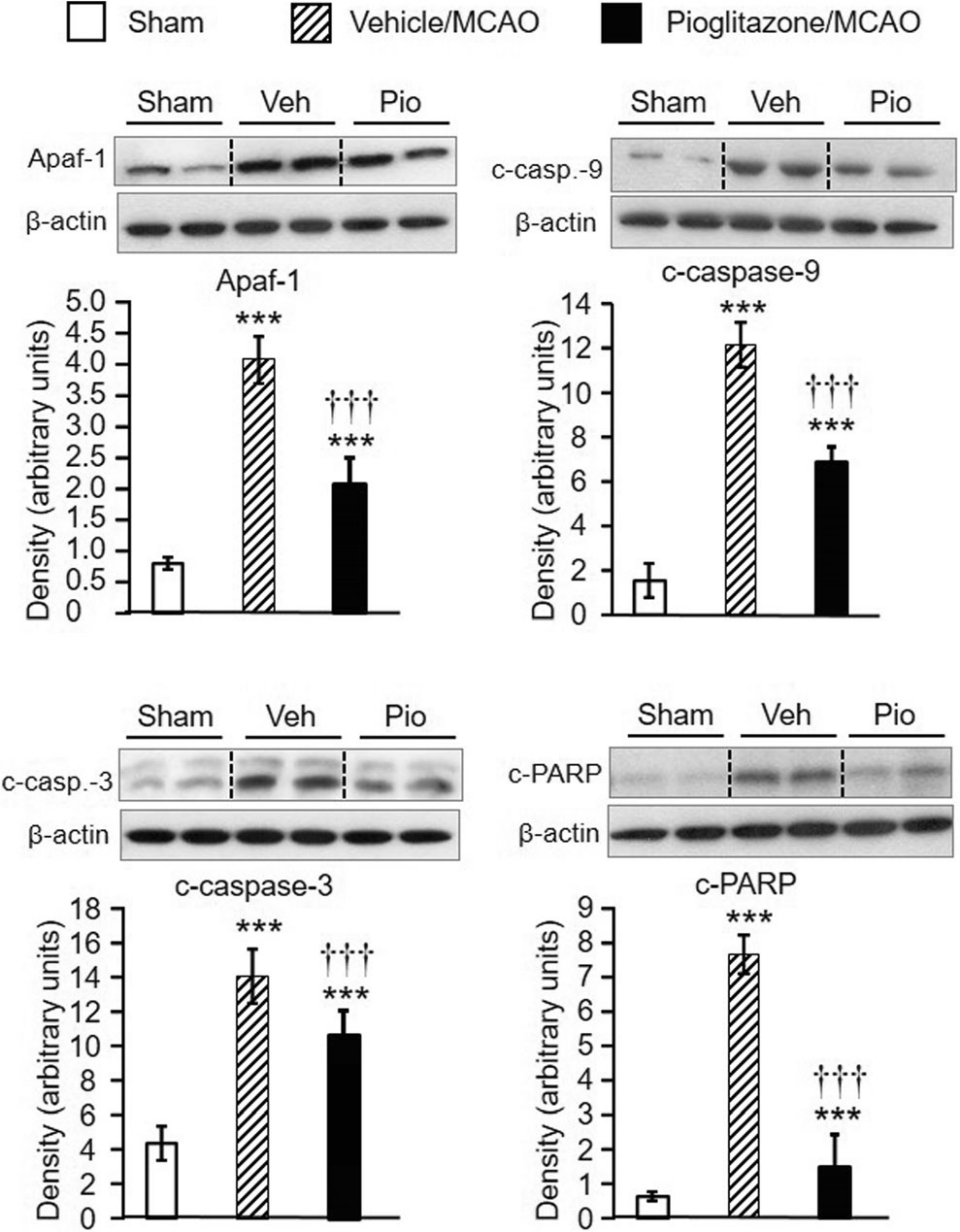
Western blot analysis of Apaf-1 (apoptotic protease-activating factor-1), c-caspase-9 (cleaved caspase-9), c-caspase-3 (cleaved caspase-3), and c-PARP (PARP p85 fragment) in the frontoparietal cortex of sham-operated rats (*n* = 6–7) and in the peri-infarct frontoparietal cortex isolated 24 h after MCAO in rats treated intracerebroventricularly with vehicle (*n* = 6–7) or pioglitazone (*n* = 6–7). Representative blots and graphical analysis are shown for each parameter. Results are expressed as the means ± SD. All apoptosis parameters were substantially upregulated 24 h after MCAO and pioglitazone effectively reduced their induction. ***P<0.001, statistical comparison to sham-operated rats and ^†††^*P*<0.001, statistical comparison of the pioglitazone-treated to the vehicle-treated groups of rats subjected to MCAO, calculated by one-way ANOVA followed by a post hoc Bonferroni test

**Fig. 3:**
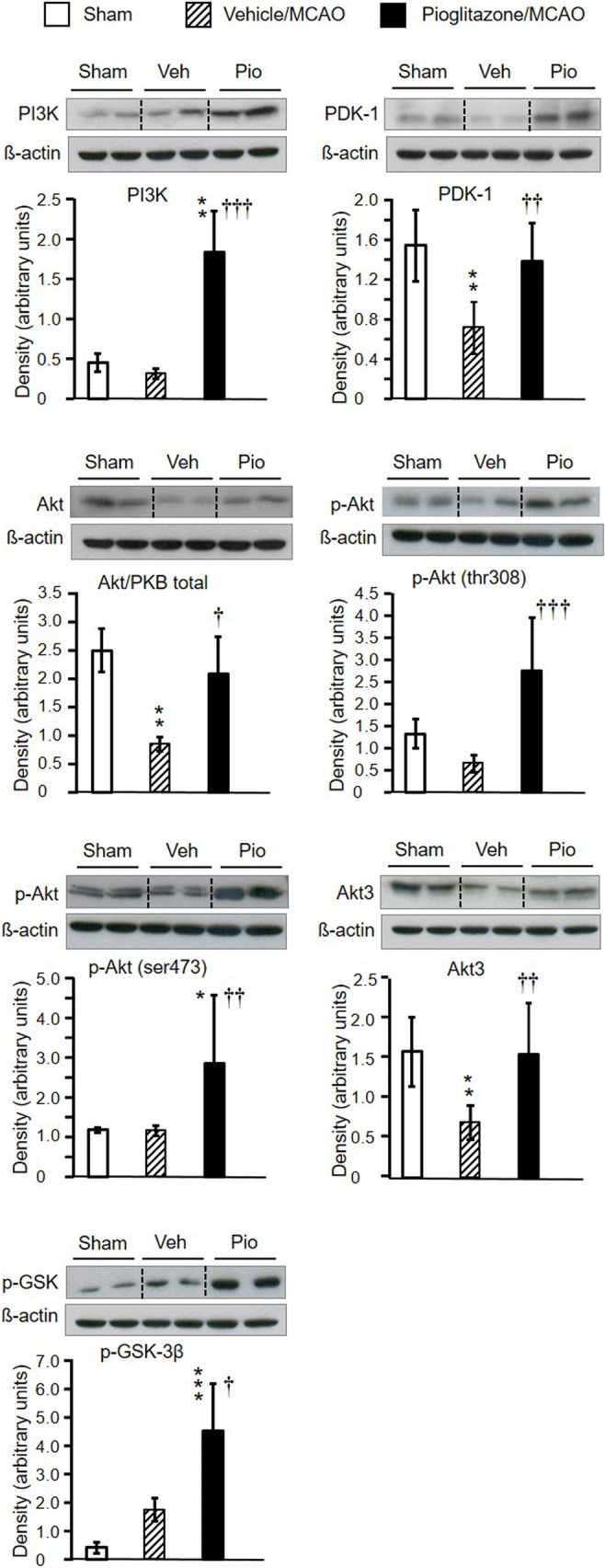
Western blot analysis of PI3K (phosphoinositide 3-kinase), PDK-1 (phosphoinositide-dependent kinase-1) Akt/PKB (protein kinase B), p-Akt (thr308) (phosphorylated Akt at threonine 308), p-Akt (ser473) (phosphorylated Akt at serine 473) Akt 3, and p-GSK-3β (ser9) (phosphorylated glycogen synthase kinase-3β at serine 9), in the frontoparietal cortex of sham-operated rats (*n* = 6–7) and the peri-infarct frontoparietal cortex isolated 48 h after MCAO in rats treated intracerebroventricularly with vehicle (*n* = 6–7) or pioglitazone (*n* = 6–7). Representative blots and graphical analysis are shown for each parameter. Results (arbitrary units) are expressed as the means ± SD. Compared to vehicle-treated rats exposed to MCAO, pioglitazone upregulated the expression of all key proteins of the PI3K/Akt signalling pathway. *P<0.05, **P<0.01, and ***P<0.001, statistical comparison to sham-operated rats; ^†^*P*<0.05, ^††^*P*<0.01, and ^†††^*P*<0.001, statistical comparison of the pioglitazone-treated to the appropriate vehicle-treated groups of rats subjected to focal cerebral ischemia, calculated by one-way ANOVA followed by a post hoc Bonferroni test (PDK and Akt 3) or by the Kruskal-Wallis test (PI3K, total Akt/PKB, p-Akt (thr 308), p-Akt (ser 473), and p-GSK-3β (ser 9))

**Fig. 4:**
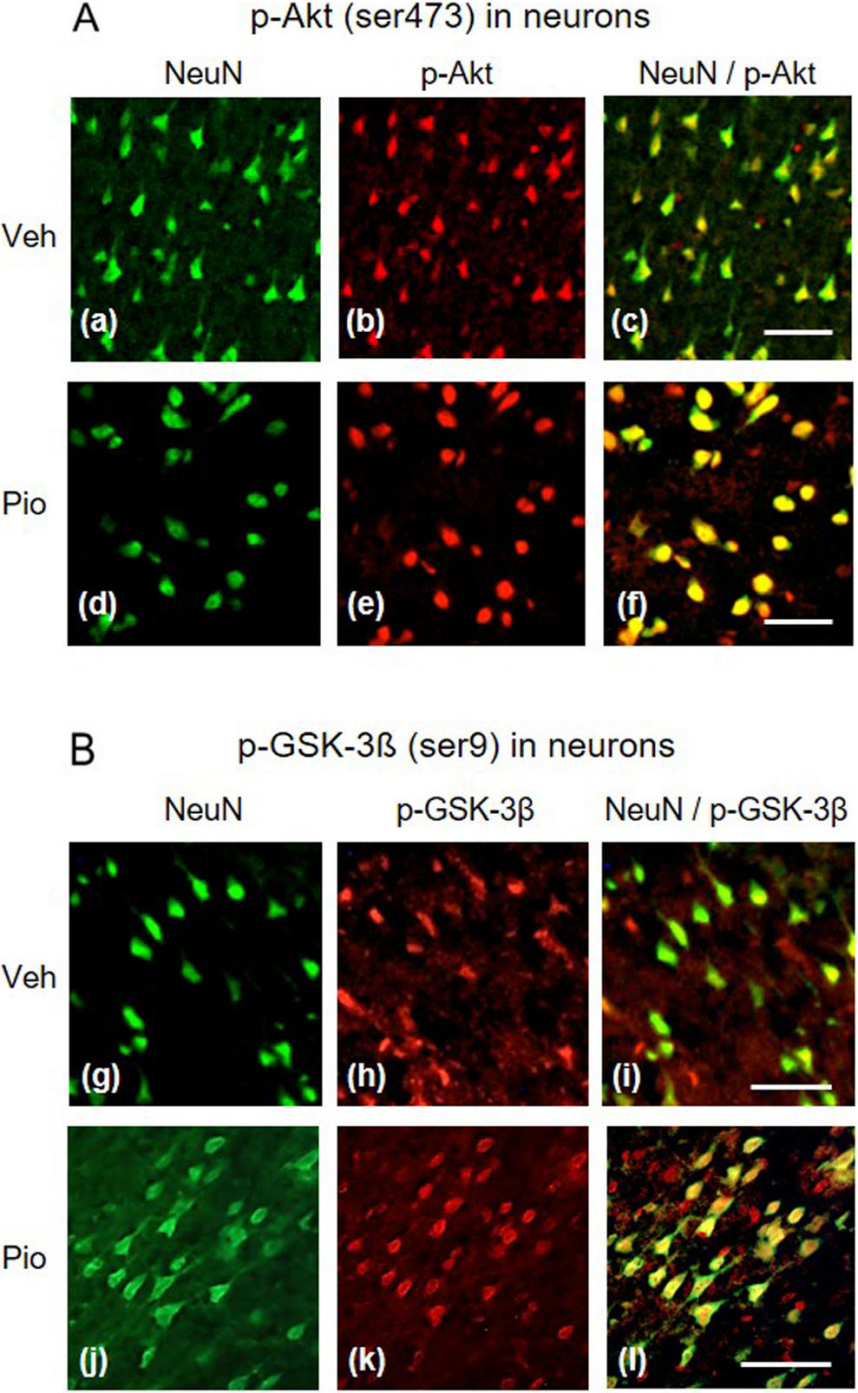
Expression of phosphorylated Akt (ser473) (p-Akt) and phosphorylated glycogen synthase kinase-3β (ser9) (p-GSK-3β) in neurons localized in the peri-infarct frontoparietal cortex isolated 48 h after MCAO. A Immunofluorescence staining against NeuN (neurons: a, d) (green) and p-Akt (b, e) (red). Overlapping of p-Akt immunoreactivity in neurons (c, f) (yellow) shows that only a few neurons express p-Akt in vehicle-treated rats (upper panels), but a majority of neurons display a substantial induction of p-Akt in pioglitazone-treated rats (lower panels). B Immunofluorescence staining against NeuN (g, j) (green) and p-GSK-3β (h, k) (red). Overlapping of p-GSK-3β immunoreactivity in neurons (i, l) (yellow) demonstrates that a few neurons express p-GSK-3β in vehicle-treated rats (upper panels), but a majority of neurons express p-GSK3β in pioglitazone-treated rats (lower panels). Scale bar 100 μm

In vehicle-treated rats, all apoptosis markers were considerably upregulated already 24 h after MCAO. Pioglitazone effectively reduced their induction but failed to completely abolish their expression which still remained higher than that detected in sham-operated animals (apoptotic protease-activating factor-1 (Apaf-1): *F*_2,16_=130.47, *P*<0.001; cleaved (c)-caspase-9: *F*_2,16_=228.85, *P*<0.001; cleaved (c)-caspase-3: *F*_2,16_=78.24, *P*<0.001; PARP p85 fragment (c-PARP): *F*_2,17_=82.68, *P*<0.001) (Fig. 2). Compared to sham-operated animals, all apoptosis markers were still significantly upregulated 48 h, but to a lesser extent than 24 h after MCAO. Pioglitazone failed to suppress the slightly increased expression of c-caspase-9 and c-caspase-3 (c-caspase-9: sham: 1.36±0.46, vehicle/MCAO: 2.14±0.51^a^, pioglitazone/MCAO: 1.53±0.35; c-caspase-3: sham: 1.78±0.92, vehicle/MCAO: 4.92±1.33^c^, pioglitazone/MCAO: 3.62±0.36^a^; ^a^*P*<0.05 and ^c^*P*>0.001 vs sham-operated rats). However, pioglitazone downregulated the expression of Apaf-1 and c-PARP (Apaf-1: sham: 2.05±0.50, vehicle/MCAO: 3.70±0.49^b^, pioglitazone/MCAO:1.90±0.49^§^; c-PARP: 2.01±0.25, vehicle/MCAO: 11.14±2.8^c^, pioglitazone/MCAO: 7.28±0.88^c†^; ^b^*P*<0.01 and ^c^*P*<0.001, statistical comparison to sham-operated rats; ^§^*P*<0.01 and ^†^*P*<0.001, statistical comparison of the pioglitazone/MCAO group to the vehicle/MCAO group of rats). Survival of peri-infarct neurons which escaped apoptosis is driven by neuroprotective mechanisms. Twenty-four hours after MCAO, the tissue levels of all parameters of the PI3K/Akt cascade did not significantly differ among the experimental groups (Table 1). However, substantial changes in their expression were detected 48 h after ischemic damage (Fig. 3). The concentrations of PDK-1, total Akt, and Akt 3 decreased in ischemic tissue. ICV infusion of pioglitazone prevented this dramatic fall and returned the rate of their expression to values detected in sham-operated animals (PDK-1: *F*_2,17_=11.21, *P*<0.001; Akt: *P*<0.01; Akt 3: *F*_2,17_=8.01, *P*<0.01). Ischemic injury did not affect PI3K, p-Akt (thr308), p-Akt (ser473), and p-GSK-3β (ser9) levels in the peri-infarct tissue compared to sham-operated rats. Pioglitazone, however, considerably enhanced their expression (PI3K: *P*<0.001; p-Akt (thr308): *P*<0.001; p-Akt (ser473): *P*<0.01; p-GSK-3β (ser9): *P*<0.001) (Fig. 3). Double immunofluorescence staining of neurons showed a weak neuronal expression of p-Akt (ser473) and p-GSK-3β (ser9) in vehicle-treated rats. In contrast, pioglitazone augmented their expression in neurons localized at the border of the ischemic core (Fig. 4) (number of positive neurons (expressed in %) p-Akt (ser473): vehicle (*n*=4): 36±10, pioglitazone (*n*=5): 51±6, *P*<0.02; p-GSK-3β (ser9): vehicle: (*n*=5): 30±14, pioglitazone (*n*=5): 56±16, *P*<0.05).

**Table 1:** Effect of intracerebroventricular infusion of vehicle or pioglitazone on the PI3K/Akt signalling pathway in the frontoparietal cortex of sham-operated rats and in the peri-infarct frontoparietal cortex of rats 24 h after MCAO

Parameter	Sham	Vehicle/MCAO	Pioglitazone/MCAO
PI3K	1.08 ± 0.28	0.66 ± 0.56	1.03 ± 0.80
PDK-1	0.72 ± 0.27	0.58 ± 0.48	1.24 ± 0.92
Akt/PKB total	2.72 ± 0.83	1.21 ± 1.01	1.79 ± 1.23
p-Akt (thr308)	1.31± 0.38	0.73 ± 0.50	1.28 ± 1.07
p-Akt (ser473)	2.96 ± 1.33	1.72 ± 2.61	4.51 ± 3.14
Akt 3	1.66 ± 0.31	0.98 ± 0.67	1.43 ± 1.20
p-GSK-3β (ser9)	0.93 ± 1.28	1.51 ± 1.20	1.95 ± 1.12

Western blot analysis of *PI3K* (phosphoinositide 3-kinase), *PDK-1* (phosphoinositide-dependent kinase-1), *Akt/PKB* (protein kinase B), *p-Akt (thr308)* (phosphorylated Akt at threonine 308), *p-Akt (ser473)* (phosphorylated Akt at serine 473), Akt 3, and *p-GSK-3β (ser9)* (phosphorylated glycogen synthase kinase-3β at serine 9), in the frontoparietal cortex of sham-operated rats (*n* = 6–7) and in the peri-infarct frontoparietal cortex isolated 24 h after MCAO in rats treated intracerebroventricularly with vehicle (*n* = 6–7) or pioglitazone (*n* = 6–7). Results (arbitrary units) are expressed as the means ± SD. Statistical comparisons revealed no significant differences in the expression of all key proteins of the PI3K/Akt signalling pathway among the experimental groups of rats (one-way ANOVA)

**Fig. 5:**
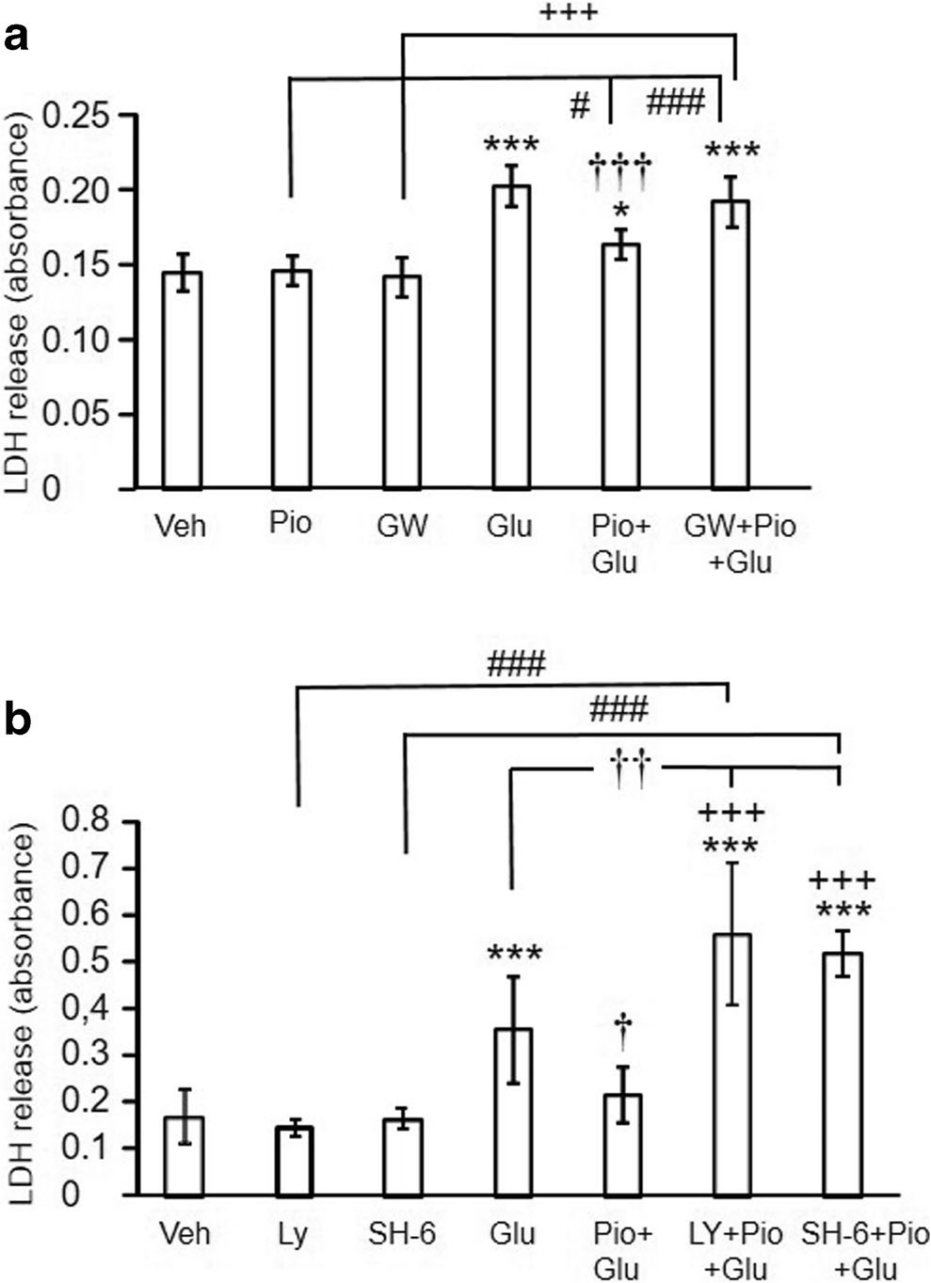
A Effects of pioglitazone (Pio) and GW 9662 (GW) on neuronal damage induced by glutamate (Glu) in primary neuronal cells. Pioglitazone abolished the glutamate-induced lactate dehydrogenase (LDH) release and GW 9662 completely reversed this effect. Results are expressed as the means ± SD. *P<0.05, ***P<0.01, statistical comparison to vehicle-treated cells; ^†††^*P*<0.001, statistical comparison of cells treated with pioglitazone + glutamate to glutamate-treated cells; ^#^*P*<0.05, ^###^*P*<0.001, statistical comparison to cells treated with pioglitazone alone; ^+++^*P*<0.001, statistical comparison to cells treated with GW 9662 alone (one-way ANOVA followed by a post hoc Bonferroni test). B Effects of the potent phosphoinositide kinase inhibitor, LY294002 (Ly) and the selective Akt inhibitor SH-6 on the glutamate-induced neuronal damage in primary neuronal cells pre-treated with pioglitazone. Both inhibitors not only completely reversed the beneficial effect of pioglitazone, they even augmented the effect of glutamate on LDH release. Results are expressed as the means ± SD. ***P<0.001, statistical comparison to vehicle-treated cells; ^†^*P*<0.05, ^††^*P*<0.01, statistical comparison to glutamate-treated cells; ^+++^*P*<0.001, statistical comparison to pioglitazone + glutamate-treated cells; ^###^*P*<0.001, statistical comparison of Ly294002-treated to Ly294002 + pioglitazone + glutamate-treated cells and of SH-6-treated to SH-6 + pioglitazone + glutamate-treated cells (one-way ANOVA followed by a post hoc Bonferroni test)

The direct effect of PPARγ on PI3K and Akt signalling was investigated in rat primary cortical neurons exposed to glutamate for 24 h. As reported previously [15], increased LDH release induced by a 24-h exposure of neurons to glutamate was attenuated by prior treatment with pioglitazone. The selective PPARγ antagonist GW 9662 reversed the pioglitazone-induced inhibition of LDH release (Fig. 5A). In the second experiment, the PI3K inhibitor LY294002 or the Akt inhibitor SH-6 potentiated the effect of glutamate on LDH release (Fig. 5B).

### Pioglitazone augments the expression of Nrf2 and HO-1 in the peri-infarct frontoparietal cortex

**Fig. 6:**
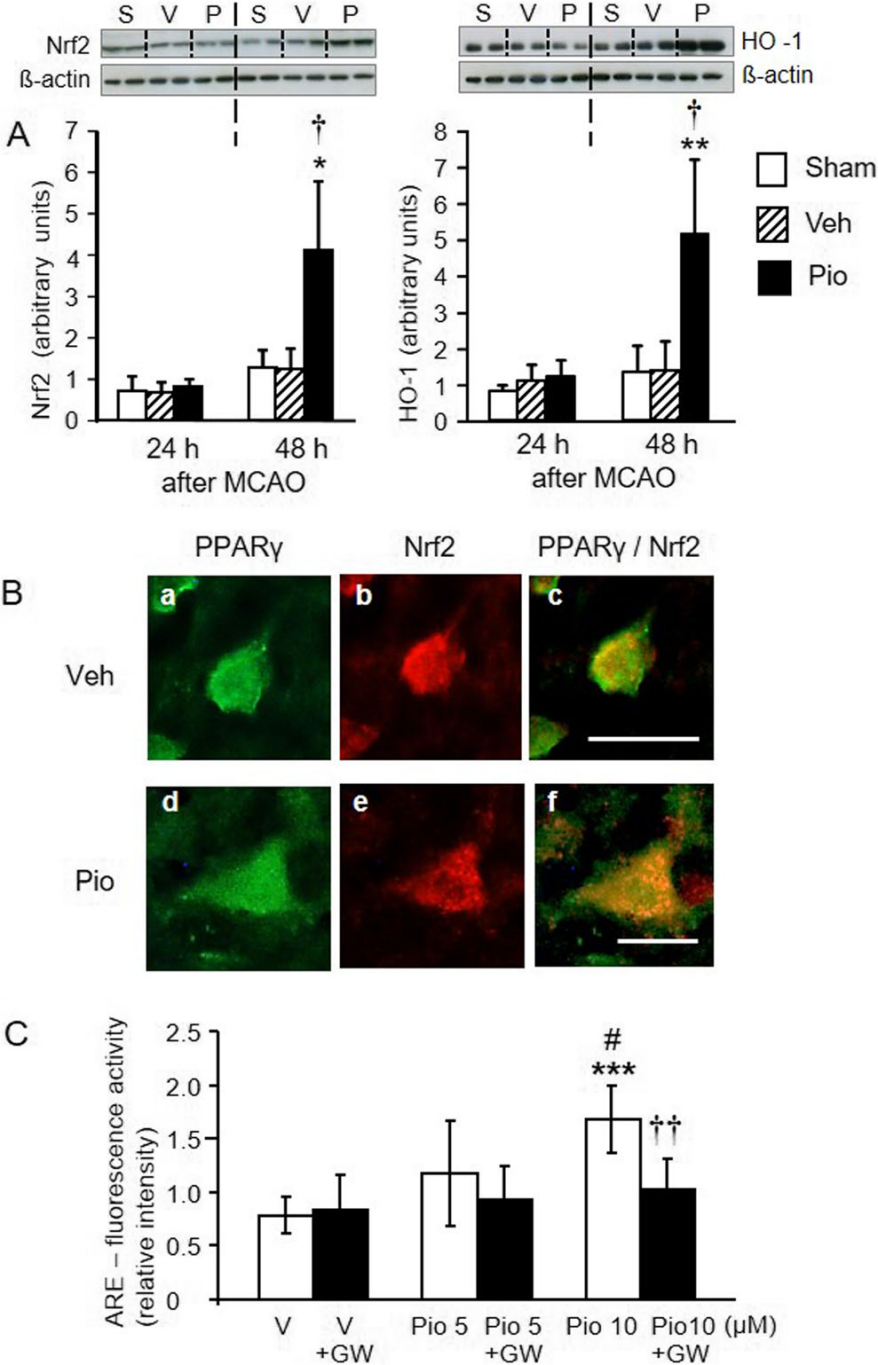
A Effects of pioglitazone (Pio) on the expression of Nrf2 (left panel) and heme oxygenase-1 (HO-1) (right panel) in the peri-infarct frontoparietal cortex 24 h and 48 h after MCAO. Pioglitazone considerably increased the expression of Nrf2 and HO-1 48 h after MCAO, when compared with the sham-operated or vehicle (Veh)-treated groups of rats. Representative blots and graphical analysis are shown for each parameter (S, sham; V, vehicle-treated; P, pioglitazone-treated rats). Results are expressed as the means ± SD. *P<0.05; **P<0.01, statistical comparisons with sham-operated rats and ^†^*P*<0.05, statistical comparison with the vehicle-treated group of rats exposed to MCAO, calculated by Kruskal-Wallis test. B Immunofluorescence staining for PPARγ (a, d) (green) and Nrf2 (b, e) (red) in neurons localized at the border of the ischemic core in vehicle (Veh)-treated (upper panels) and pioglitazone (Pio)-treated (lower panels) rats. Overlapping of PPARγ and Nrf2 immunoreactivities (c, f) (yellow) shows a considerable induction of Nrf2 in pioglitazone-treated rats. Scale bar: c 50 μm, f 25 μm. C PC12 cells were transfected with reporter construct pNQO1-r antioxidant response element (ARE) plasmid and exposed to vehicle (V) or pioglitazone (Pio). Pioglitazone activated ARE and this effect was completely abolished by GW 9662 (GW). Results are expressed as the means ± SD. ***P<0.001 statistical comparison with vehicle-treated cells; ^#^*P*<0.05, statistical comparison with cells exposed to 5 μM pioglitazone. ^††^*P*<0.01, statistical comparison with cells treated with 10 μM pioglitazone, calculated by one-way ANOVA followed by a post hoc Bonferroni test

In addition to PI3K/Akt signalling, pioglitazone activated another neuroprotective pathway comprising the Nrf2 transcription factor and its target gene, HO-1. Pioglitazone induced a robust Nrf2 and HO-1 expression in the peri-infarct cortical tissue 48 h after the ischemic insult (Fig. 6A). In neurons localized directly at the border of the ischemic core, pioglitazone considerably upregulated the expression of the Nrf2 already 24 h after MCAO (Fig. 6B).

### Induction of ARE-mediated reporter activity by pioglitazone

PC12 cells were transfected with the constructed reporter pNQO1-rARE luciferase plasmid and luciferase activity was measured 24 h after incubation of cells with 1, 5, or 10 μM pioglitazone in the presence or absence of the PPARγ receptor antagonist GW 9662 (1 μM). Pioglitazone dose-dependently activated the luciferase gene expression. One micromolar pioglitazone had no effect (data not shown), but 5 and 10 μM pioglitazone activated the luciferase gene expression 1.5-fold (not significantly vs vehicle) and 2.2-fold (*P*<0.001 vs vehicle), respectively. Ten micromolar pioglitazone activated the luciferase gene expression more effectively than 5 μM (*P*<0.05). The effect of pioglitazone was completely antagonized by GW 9662 (*F*_5,42_=6.96, *P*<0.001) (Fig. 6C).

### Pioglitazone requires Nrf2 to protect neurons against oxidative damage and excitotoxicity

**Fig. 7:**
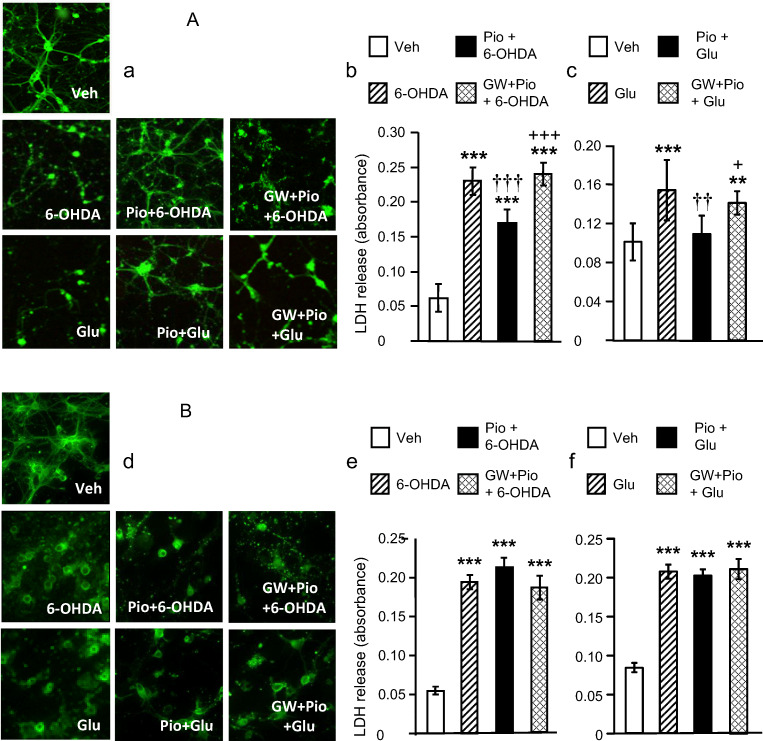
Neuroprotective and neuroregenerative effects of pioglitazone (Pio) in mouse primary cortical neurons exposed to oxidative and excitotoxic damage induced by 6-hydroxydopamine (6-OHDA) and glutamate (Glu), respectively, are Nrf2-dependent. Control mice (A), left panels (a): Immunofluorescence staining against β III-tubulin (green) shows that primary cortical neurons treated with vehicle developed neuritic and synaptic networks. Neurons exposed to 6-OHDA or Glu retained neurites and displayed serious neuronal damage. Pioglitazone restored the formation of synaptic networks in neurons exposed to 6-OHDA or Glu. The PPARγ antagonist, GW 9662 (GW), completely reversed the neuroprotective and neuroregenerative effects of pioglitazone. Middle panel (b) and right panel (c): Pioglitazone suppressed the 6-OHDA- or Glu-induced lactate dehydrogenase (LDH) release in primary cortical neurons of control mice and GW 9662 reversed this effect. Results are expressed as mean ± SD. **P<0.01, ***P<0.001, statistical comparison to neurons treated with vehicle; ^††^*P*<0.01 and ^†††^*P*<0.001 statistical comparison to neurons treated with Glu and 6-OHDA, respectively; ^+^*P*<0.05 and ^+++^*P*<0.001, statistical comparison to Glu + pioglitazone-treated neurons and to 6-OHDA + pioglitazone-treated neurons, respectively, calculated by one-way ANOVA followed by a post hoc Bonferroni test. Nrf2 knockout mice (B), left panel (d): Pioglitazone failed to protect primary cortical neurons obtained from Nrf2 knockout mice against oxidative and excitotoxic damage induced by 6-OHDA or Glu. Middle panel (e) and right panel (f): Pioglitazone did not suppress the LDH release induced by 6-OHDA or Glu in these neurons. ***P<0.001, statistical comparison to vehicle-treated neurons, calculated by one-way ANOVA followed by a post hoc Bonferroni test

The development of synaptic networks, characterized by the outgrowth of neurites and dendrites, is intimately associated with β III-tubulin (Fig. 7A, upper panel [a]) [17, 18]. Neurons from wild-type mice exposed to 6-OHDA or glutamate died or displayed signs of severe damage, characterized by the retraction and loss of neurites and dendrites. Pioglitazone promoted neurite outgrowth and formation of synaptic networks even in neurons exposed to 6-OHDA or glutamate and this effect was completely reversed by GW 9662, clearly indicating a PPAR gamma-dependent mechanism (Fig. 7A, middle and lower panels [a]). The exposure of neurons to 6-OHDA or glutamate increased LDH release. Pioglitazone reduced the LDH release, and again, this rescue effect was completely abolished by GW 9662 (6-OHDA: *F*_3,20_=366.05, *P*<0.001; glutamate: *F*_3,32_=11.88, *P*<0.001) (Fig. 7A [b, c]). Most importantly, pioglitazone failed to protect primary cortical neurons from Nrf2 knockout mice against oxidative and excitotoxic damage (Fig. 7B [d, e, f]).

## Discussion

Previous findings have demonstrated that pioglitazone in the brain interacts with a number of pathophysiological events which evolve in ischemic brain tissue. In the early phase after ischemic stroke, pioglitazone reduces inflammatory reactions [7, 9, 10], which along with neuronal cell death substantially contribute to the expansion of ischemic injury. The present study demonstrates that pioglitazone acting on cerebral PPARγ exerts concerted actions resulting in inhibition of apoptosis and activation of neuroprotective cascades, including the Nrf2/ARE pathway in neurons adjacent to the ischemic core. The most prominent finding is the unique feature of pioglitazone which, besides the Nrf2 upregulation, also augments its binding to the ARE to increase the ARE activity.

The outcome after ischemic stroke depends on the interplay between apoptotic and anti-apoptotic events in the penumbra. Pioglitazone-infused ICV alleviated motor and sensory deficits more effectively 24 h after MCAO and suppressed the apoptotic cascade, including the activation of the executioner caspase-3, an indicator of the irreversible progression of apoptosis. The underlying mechanisms are not fully understood. Anti-apoptotic effects of the PPARγ agonists 15d-PGJ_2_ and rosiglitazone, comprising the suppression of H_2_O_2_ generation and cytochrome-c release from mitochondria, have been demonstrated in an in vitro study [19].

The activation of the neuroprotective PI3K/Akt pathway supporting survival of peri-infarct neurons is essential for the improved recovery from ischemic stroke. Akt bound to the cell membrane via PIP3 synthesized by PI3K is activated by phosphorylation of threonine 308 and serine 473. The Akt-mediated phosphorylation promotes neuronal survival [11, 20–22]. GSK-3β acts as pro-apoptotic by activation of the pro-apoptotic factors, e.g., p53, and promotion of Nrf2 degradation [21, 22]. GSK-3β phosphorylation of serine 9 by Akt inactivates the kinase and prevents thus neuronal apoptosis [11]. Following cerebral ischemia, p-Akt is dephosphorylated and inactivated, a tendency also seen in the present study. A sustained phosphorylation of Akt was reported in the time period from 24 to 72 h after ischemic injury [21]. We report here that pioglitazone reversed the ischemia-induced downregulation of Akt and increased p-Akt (thr308) and p-Akt (ser473), particularly 48 h after cerebral ischemia. A substantial upregulation of p-GSK-3β (ser9) was also detected at the same time point. The augmented expression of p-Akt (ser473) and p-GSK-3β (ser9) occurred in severely damaged neurons localized in close proximity to the ischemic core indicating that the upregulation of both kinases is prominent in neurons in which the impact of ischemia is most pronounced. Furthermore, inhibitors of PI3K or p-Akt completely antagonized the pioglitazone-mediated neuroprotection in primary cortical neurons severely affected by neurotoxicity. All these findings convincingly demonstrate that interaction of pioglitazone with cerebral PPARγ considerably contributes to the activation of the PI3K/Akt pathway, which is considered a fundamental factor of neuronal survival after ischemic injury.

Oxidative stress is the hallmark of the response of brain tissue to ischemia. Excessive production of ROS leads to transcriptional activation of antioxidant genes initiated by binding of Nrf2 to the ARE [23]. Oxidative stress, Nrf2 phosphorylation, or GSK-3β inactivation promotes the translocation of Nrf2 into the nucleus and its binding to the ARE induces expression of antioxidant genes [24–27]. There are multiple interactions between the PPARγ and Nrf2 in terms of the regulation of the antioxidant gene expression. As the Nrf2 gene contains the peroxisome proliferator-activated receptor response elements (PPRE), activated PPARγ upregulates Nrf2, which, in turn, enhances the PPARγ expression. The localization of the PPRE and ARE in antioxidant genes allows their synergistic activation by both PPARγ and Nrf2 [28].

Intense Nrf2 immunoreactivity in ischemic neurons was already detected early, 24 h after cerebral ischemia (present data and [29]). In contrast, Western blotting revealed a substantial rise in Nrf2 concentration in the peri-infarct cortical tissue at 48 h. Immunofluorescence staining shows neurons severely affected by ischemia. On the other hand, Nrf2 quantification by Western blotting was carried out in a strip of peri-infarct brain tissue containing both severely damaged tissue and areas only moderately affected by blood flow restrictions. The induction of the Nrf2/ARE system in ischemic tissue was confirmed by the upregulation of HO-1. Apart from the Nrf2 upregulation, we demonstrate for the first time that pioglitazone also dose-dependently induces activation of the Nrf2/ARE system and thereby enhances the expression of a number of genes encoding for antioxidative enzymes indicating a robust activation of the antioxidant defense pathway in ischemic brain tissue.

Substantial evidence for the essential role of Nrf2 in the pioglitazone-mediated protection of neurons against oxidative stress or excitotoxicity is provided by experiments employing primary cortical neurons obtained from control and Nrf2 knockout mice. Pioglitazone prevented cell death induced by 6-OHDA or glutamate in cortical neurons from control mice by a PPARγ-dependent mechanism. The underlying mechanisms may involve the activation of antioxidant genes by the Nrf2/ARE system as pioglitazone failed to protect cortical neurons from Nrf2 knockout mice against oxidative and excitotoxic damage. This finding underlines the essential role of Nrf2 in the pioglitazone-triggered activation of the antioxidant defense pathway in injured neurons and prevention of neuronal death.

In conclusion, stimulation of PPARγ in ischemic brain tissue activates the neuroprotective and antioxidant defense pathways supporting the survival of viable neurons in the peri-infarct regions. The data substantiates the relevance of cerebral PPARγ for protection of neurons against ischemic damage which at last results in the improved recovery from cerebral ischemia. The results provide novel insight into the neuroprotective mechanisms linked to the activation of cerebral PPARγ and the rationale for conducting additional clinical trials investigating the use of pioglitazone in acute treatment of patients suffering from ischemic stroke [30].

## Supplementary Information

ESM 1 (DOCX 25.2 kb)

## Declarations

### Data transparency

All authors confirm that all data and material support their published claims and comply with field standards.
