# Supplementary material for: Neuroprotective and antioxidative effects of pioglitazone in brain tissue adjacent to the ischemic core are mediated by PI3K/Akt and Nrf2/ARE pathways
Source: J Mol Med (Berl). 2021 Apr 16;99(8):1073–83. doi: 10.1007/s00109-021-02065-3 (PMC8313471; doi:10.1007/s00109-021-02065-3)
Supplement: Supplementary file 1 — (DOCX 25.2 kb) [file 109_2021_2065_MOESM1_ESM.docx]

**The Supplement**

*Neurological outcome*

Neurological evaluations were carried out 24 h or 48 h after MCAO by the same investigator, who was not involved in surgery and was blinded to the treatment of rats. The neurological grading system developed by Garcia et al [14] was used. This grading system allows a separate assessment of the following motor and sensory functions: 1. spontaneous activity, 2. symmetry of the movement of four limbs, 3. forepaw outstretching, 4. climbing, 5. body proprioception and 6. response to vibrissae touch. Severe impairment in each test are graded 0 or 1, no observable deficits are grade 3. The individual score of each rat is the summation of all six individual test scores. The minimum of the total neurological score is 3, the maximum is 18 [14].

*Western blot analysis of parameters linked to apoptosis and neuroprotection*

The proteins from the dissected brain areas were isolated [7] and their concentrations were determined using the BCA^TM^ protein assay Kit (Pierce, Rockford, IL, USA). Equivalent amounts of total proteins (15 or 30 µg per sample) were loaded and separated on 10% or 12% SDS-polyacrylamide gels and transferred to polyvinylidene difluoride transfer membranes (Millipore Corporation). After washing with Tris-buffered saline containing 0.1% Tween-20 (TTBS) and blocked with 4% non-fatty dry milk, the membranes were incubated with the following primary polyclonal (p) or monoclonal (m) antibody (Ab) at 4°C overnight:
Apoptosis-parameter: rabbit anti APAF-1 (apoptotic protease activating factor -1) pAB (1:1000, Cell Signaling Technology, Cat-Nr. 4452), rabbit anti c-caspase-9 (cleaved caspase 9) pAB (1:1000, Cell Signaling Technology, Cat-Nr. 9507), rabbit anti c-caspase-3 (cleaved caspase-3) pAB (1:1000, Cell Signaling Technology, Cat-Nr. 9664), and rabbit anti c-PARP (PARP p85 fragment) pAb (1:1000, Promega, Cat-Nr. G734);
Components of the PI3K/Akt pathway, Nrf2 and heme-oxidase-1: mouse anti PI3K mAb (1:3000, BD Biosciences, Cat-Nr. 610045), rabbit anti PDK-1 pAb (1:3000, Cell Signaling Technology, Cat-Nr. 3062), rabbit anti Akt1/2/3 pAb (1:5000, Santa Cruz Biotechnology, Cat-Nr. sc-8312), mouse anti phospho-Akt (ser473) mAb (1:3000, Cell Signaling Technology, Cat-Nr. 4051), rabbit anti phospho-Akt (thr308) pAb (1:2000, Santa Cruz Biotechnology, Cat-Nr. sc-16646-R), goat anti Akt 3 pAb (1:1000, Santa Cruz Biotechnology, Cat-Nr. sc-11521), rabbit anti phospho-GSK-3ß (ser9) pAb (1:2000, Cell Signaling Technology, Cat-Nr. 9336), mouse anti Nrf2 mAb (1:1000, R&D System, Cat-Nr. MAB3925), rabbit anti heme oxidase-1 mAb (1:3000, Enzo Life Sciences, Cat-Nr. ADI-OSA-110 ), mouse anti ß-actin mAb (1:10,000, Sigma-Aldrich, Cat-Nr. A 5441). After three washing steps with TTBS, the membranes were incubated with horseradish peroxidase-conjugated goat anti-rabbit or anti-mouse secondary antibody (1: 3000, Amersham Biosciences), or donkey anti goat secondary antibody (1: 5000, Santa Cruz Biotechnology) for 30 min at room temperature (RT). The signal was visualised using the enhanced chemiluminescence (ECL) detection system and high-performance film. The detected signal was identified with the molecular weight of the corresponding protein. ß-actin was used as a loading control.

*Immunofluorescence staining for phospho-Akt (ser473), phospho-GSK-3β (ser9), PPARγ and Nrf2 in neurons localised at the border of the infarct region* Serial 10 µm coronal sections were washed with PBS containing 0.2 % Triton (PBST), blocked with 1% of bovine serum albumin (BSA) and incubated with the first primary Ab, rabbit anti phospho-Akt (ser473) pAb (1:500, Cruz Biotechnology, Cat-Nr. sc-7985-R ) or rabbit anti phospho-GSK-3ß (ser9) pAb (1:300, Cell Signaling Technology, Cat-Nr. 9336), rabbit anti PPARγ (1:200, Santa Cruz Biotechnology, Cat-Nr. sc-7196) at 4°C overnight. For double immunofluorescence staining, the sections were incubated with the second primary Ab, mouse mAb against neuronal nuclei (NeuN) (1: 100, Merck, Cat-Nr. MAB 377) or mouse anti Nrf2 mAb (1:100, R&D System, Cat-Nr. MAB3925) at 4°C for 4 h. After washing, the brain sections were incubated with the corresponding secondary Ab in 1 % BSA at RT for 1 h as follows: Alexa Fluor488®-conjugated donkey anti-mouse Ab (staining for NeuN or Nrf2), or Alexa Fluor546®-conjugated goat anti-rabbit Ab (staining for phospho-Akt (ser473) or phosphor-GSK-3ß (ser9) or PPARγ (Thermo Fisher Scientific). After washing in PBS, the sections were covered in ProLong™ Gold Antifade Mountant Reagent (Molecular Probes, Leiden, The Netherlands). The stained sections were analyzed with a Leica DMR fluorescence microscope and phospho-Akt (ser473) - and phospho-GSK-3β (ser9)-positive neurons were counted.

Primary neuronal cell culture

The cerebral cortices from 0 to 1 day neonatal Wistar rats, wild-type (C57BL/6) mice or Nrf2 knock-out mice were dissected and dispersed using 0.25 % trypsin followed by gentle trituration to release cells. After washing with neurobasal medium (Invitrogen GmbH, Darmstadt, Germany), the neuronal cells were counted and plated onto L-poly-lysine pre-coated four-well plates. For the immunofluorescence staining, cells were plated on cover slides in four-wells plates with L-poly-lysine pre-coated cover slides (1.5 x 10^5^/well), and for the LDH assays, cells were plated at a density of 2 x 10^5^ per well in four-well plates. Cells were maintained in the neurobasal medium containing 2 % B27 supplement, 0.5 mM L-glutamine, 100 U/ml penicillin, 100 µg/ml streptomycin (Invitrogen GmbH, Darmstadt, Germany) in a humidified atmosphere of 5 % CO2/95 % air at 37 °C. Every three days, half of the culture medium was changed. Seven days after plating, primary cortical neurons were used for the intended experiments.

Table A Effects of pioglitazone, GW 9662, PI3K inhibitor, LY294002, and Akt –inhibitor, SH-6, on the cytotoxicity in rat primary cortical neurons (release of lactate dehydrogenase)

Substance Vehicle Concentration (µM)

 1 5 10

Pioglitazone 0.145 ± 0.003 0.137 ± 0.003 0.140 ± 0.002 0.146 ± 0.001

GW 9662 0.142 ± 0.013 0.145 ± 0.003 0.154 ± 0.003 0.143 ± 0.002

LY294002 0.135 ± 0.003 0.142 ± 0.002 0.147 ± 0.003 0.146 ± 0.009

SH-6 0.142 ± 0.004 0.139 ± 0.002 0150 ± 0.008 0.157 ± 0.004

Rat primary cortical neurones were exposed to vehicle (n=5) or various concentrations of pioglitazone, GW 9662, LY294002, SH-6 (n=5) for 24 h and the release of lactate dehydrogenase into the culture medium was quantified. Results (absorbance) are expressed as the means ± SD. The differences in the release of lactate dehydrogenase into the culture medium between various concentrations of the tested substances and the corresponding vehicle-treated groups were not statistically significant (one-way ANOVA).

*Cell culture and transfection*

PC12 rat pheochromocytoma cells, obtained from the German Collection of Microorganisms and Cell Cultures (Braunschweig, Germany), were grown on collagen A (1:6) pre-treated dishes in DMEM supplemented with 10% horse serum, 5% fetal calf serum and 1 % penicillin/streptomycin at 37 C° in a humidified atmosphere of 5% CO_2_ /95% air. The medium was changed between two or three times per week depending on the state of the cell confluence.
 Transfection of PC12 cells and dual luciferase assay was carried out according to the method reported by Wruck et al. (2008) [16]. Both strands of the rat *NQO1* gene ARE1 5′-CAGTCTAGAGTCACAGTGACTTGGCAAAATCG-3′ and 5′-CTAGCGATTTTGCCAAGTCACTGTGACTCTAGACTGGTAC-3′ with Kpn1 and Nhe1 ends were synthesized by TIB Molbiol (Berlin, Germany). The oligonucleotides were then annealed and cloned at the Kpn1 and Nhe1 site of the pGL3-Promoter (Promega) to produce the reporter construct pNQO1-rARE plasmid containing the firefly luciferase reporter gene. The NQO1-ARE reporter (1.5 µg) containing the firefly luciferase reporter gene and pRL-TK plasmid (0.5 µg) containing the *Renilla reniformis* luciferase gene were co-transfected into PC12 cells under the control of the herpes simplex virus thymidine kinase promoter by the lipotransfection method (Lipofectamine 2000; Invitrogen) [16].

*The immunofluorescence detection of β III-tubulin in mouse primary cortical neurons from control- and Nrf2 knock-out mice* For immunofluorescence detection of β III-tubulin (neuronal), primary cortical neurons were plated on cover slides in four-well plates (1.5 x 10^5^ per well). Twenty four h after treatment with vehicle, or pioglitazone with the presence or absence of GW 9662 and exposure to 6-OHDA (50 µM) or glutamate (100 µM), neurones were fixed wit 4 % of PFA for 1h at room temperature. The cells were then washed 3 times with PBST permeabilised with 0.1 % Triton-citrate solution for 5 min, blocked with 3 % normal goat serum for 30 min and incubated with mouse anti β III-tubulin mAb specific for neurons (1:3000, Sigma Aldrich) at 4 °C. On the next day (24 h later) neurones were incubated with Alexa Fluor® 488-conjugated goat anti mouse secondary (Molecular Probes, Leiden, the Netherlands), washed 3 times with PBS, dried in dark and mounted in Prolong® Gold antifade reagent for fluorescence microscopy analysis.
